# Greeting

**DOI:** 10.31662/jmaj.s0002

**Published:** 2018-09-28

**Authors:** Morito Monden

**Affiliations:** 1 The Japanese Association of Medical Sciences

Please allow me to make the following address for the launch of the JMA Journal.

The Asian Medical Journal (later renamed JMAJ), the one preceding the JMA Journal, was started in 1958 by Taro Takemi, a former president of the Japan Medical Association (JMA), who recognized the importance of professional exchanges in the medical field between Japan and various other countries including those of the Asian region. The Asian Medical Journal aimed to introduce medical care as practiced in Japan, the beliefs and activities of the JMA, as well as state-of-the art medical sciences to other countries, and was distributed mostly over the Asian region. It is quite remarkable that transmission of information targeting areas outside Japan was initiated in as early as 1958.

Taking over this mission, the newly produced JMA Journal consists of a wide range of submitted articles describing and documenting studies encompassing the fields of medicine and medical policies, as well as opinions and other relevant topics. All articles are published following a thorough and strict review of submitted manuscripts.

A decrease in the number of scientific articles in Japan has been pointed out for some years. It is desirable that many manuscripts be submitted by various researchers and medical experts for the sake of improving the quality of scientific research and information in Japan.

My hope is that the JMA Journal will become Japan's representative general medical journal leading other scientific journals in Japan and that it will expand to serve as an international electronic journal of science information prevailing throughout the global community.

Morito Monden, MD, PhD

President, The Japanese Association of Medical Sciences

